# Efficacy of teriparatide in the treatment of nontraumatic osteonecrosis of the femoral head: a retrospective comparative study with alendronate

**DOI:** 10.1186/s12891-016-1379-y

**Published:** 2017-01-19

**Authors:** Ryuta Arai, Daisuke Takahashi, Masahiro Inoue, Tohru Irie, Tsuyoshi Asano, Takuya Konno, Mohamad Alaa Terkawi, Tomohiro Onodera, Eiji Kondo, Norimasa Iwasaki

**Affiliations:** 1 Department of Orthopaedic Surgery, Hokkaido University Graduate School of Medicine, Kita-15, Nish-7, Kita-ku, Sapporo, 060-8638 Japan; 2 Department of Orthopaedic Surgery, Wajo Eniwa Hospital, Koganechuo 2-1-1, Eniwa, 061-1149 Japan; 3 Department of Advanced Therapeutic Research for Sports Medicine, Hokkaido University Graduate School of Medicine, Kita-15, Nish-7, Kita-ku, Sapporo, 060-8638 Japan

**Keywords:** Nontraumatic osteonecrosis of the femoral head, Teriparatide, Collapse of the femoral head

## Abstract

**Background:**

Collapse of the femoral head associated with nontraumatic osteonecrosis (NOFH) is one of the most common causes of disability in young adult patients. Excessive bone resorption by osteoclast coincident with the suppression of osteogenesis are believed to be responsible for collapse progression. Alendronate that inhibits bone resorption by inducing osteoclast apoptosis has been traditionally used for treating NOFH; however, several reports documented serious complications by the use of this drug. On the other hand, teriparatide activates osteoblasts leading to an overall increase in bone volume, and is expected to reduce the progression of femoral head collapse in NOFH. Therefore, the present study was undertaken to examine pharmacological effects of teriparatide on collapse progression of NOFH and to compare these effects with alendronate.

**Methods:**

We conducted a retrospective study in our facility for comparing the pharmacological effects of teriparatide and alendronate on 32 NOFH patients diagnosed with osteoporosis. Between 2007 and 2013, patients were treated with daily administration of 20 μg teriparatide (15 patients: 18 hips), or with 35 mg of alendronate once a week (17 patients: 22 hips). The mean period of follow-up was 18.7 months. The progression of collapse was evaluated prior to the administration and later every three months by anteroposterior radiographs. Collapse progression with > 1 mm was defined as advanced collapse, while with < 1 mm was defined as stable radiologic disease. Student’s t-test and the chi-square test was used to do compare the pharmacological effects of the two groups.

**Results:**

Treatment with terparatide had a tendency to reduce the rate of advanced collapse as compared to that with alendronate (*p* = 0.105). Kaplan-Meier curves related to stable radiologic disease showed that teriparatide-treated patients had better stable states than these treated with alendronate (*p* = 0.08, log-rank test). Moreover, treatment with teriparatide resulted in a significant reduction in collapse progression as compared to that with alendronate, noted at the end of follow-up period (*p* = 0.049).

**Conclusion:**

The present study suggests that teriparatide has greater pharmacological effects than alendronate for treating NOFH and preventing the collapse of femoral head.

**Trial registration:**

The registration number in UMIN Clinical Trial Registry is UMIN000017582. The date of registration is May 5, 2015.

## Background

Complete collapse of the femoral head is one of the major complication of nontraumatic osteonecrosis (NOFH), resulting in dysfunction of the hip and disability in patients. Collapse of the femoral head occurs in 75% of NOFH cases within three years and in 80% of patients within four years of onset of hip pain [1, 2]. The disease is typically progressive and mainly occurs in young population, whereas and most of patients eventually require total hip replacement (THR) within three years [3–5]. In fact, 10% of THRs performed in the United States is due to NOFH [4]. The pathogenesis of NOFH remains unclear, but involves interruption of the blood supply to the femoral head leading to osteonecrosis [5]. Consequently, osteonecrosis induces osteoclastic and osteoblastic remodeling processes that cause collapse of the femoral head of the necrotic region [6–8].

Efficient pharmacological treatment that prevents bone collapse is not currently available, and therefore, identifying novel drug with potential benefits is extremely desirable. Coordinating osteoclastic and osteoblastic activities is an attractive approach and may offer good therapeutic option for NOFH. Bisphosphonates that inhibit excessive osteoclast-mediated bone resorption have proven to reduce the incidence of collapse of femoral head in osteonecrotic hip [9–11]. Nonetheless, Chen et al reported that alendronate has only minor effects on the disease progression and has failed to prevent THR in majority of patients [12]. On the other hand, teriparatide is a recombinant form of parathyroid hormone that has been used as anabolic agent for treatment of osteoporosis. Teriparatide has positive effects on osteoblast differentiation and activation leading to bone necrosis lesions repair [13–15]. In support of this concept, Jiang et al reported that teriparatide improves trabecular morphology and increases cancellous bone volume and cortical bone thickness [16, 17]. Teriparatide enhances the bone healing in osteonecrotic jaw, and reduces steroid-induced osteonecrosis of femoral head [18–21].

Although several clinical studies highlight the advantageous effects of terpiaratide for the treatment of osteonecrosis, the clinical benefit of teriparatide for NOFH has not been systematically studied. Therefore, the present study was undertaken to determine whether teriparatide could offer greater pharmacological effects than alendronate, which is known to be the traditional pharmacological option for preventing collapse progression of the femoral head.

## Methods

### Patients

Between Jan 1, 2007, and Dec 31, 2013, fifty-three patients who were diagnosed with NOFH had undergone treatment for osteoporosis; nineteen patients had received teriparatide, and thirty-four patients had received alendronate. Twenty-one patients (four patients on teriparatide treatment and seventeen patients on alendronate treatment) were excluded from this study; fifteen patients had been followed for less than 6 months. Four patients developed osteoarthritis of the hip. In two patients, the administration periods were not identified. We conducted retrospective study for these thirty-two patients. Fifteen patients (18 hips; three patients were observed in bilateral hips) received teriparatide, and seventeen patients (22 hips; five patients were observed in bilateral hips) received alendronate. The patients’ mean age was 38.7 years, with 4 male and 11 female patients, in the teriparatide group, and 46.8 years, with 9 male and 8 female patients, in the alendronate group (Table 1). The mean follow-up was 18.7 months. The radiologic stages of the patients were stages 1, 2, and 3A in the Japanese Investigation Committee (JIC) staging system, and the locations of osteonecrosis were type C-1 and C-2 in the JIC classification system [22]. Stage 1 is the phase that osteonecrosis can be detected by magnetic resonance imaging (MRI) or bone scintigram, cannot be detected by X-ray. In stage 2, demarcating sclerosis is seen without collapse of femoral head on X-ray images. Stage 3 shows collapse of the femoral head without joint-space narrowing, and is subdivided into stage 3A (less than 3 mm of collapse) and 3B (3 mm or more of collapse). Type C lesion occupies more than the medial two-thirds of the weight-bearing portion. Type C-2 lesion extends laterally to the acetabular edge, whereas type C-1 lesion does not [22].

**Table 1:** Demographic characteristics of the teriparatide and alendronate groups

	Teriparatide group	Alendronate group	*p* value
Patients	15	17	
Hips	18	22	
Mean age, years (range)	38.7 (22-59)	46.8 (19-67)	0.053
Mean body weight (kg)	59.2	59.2	0.499
Mean body mass index (kg/m^2^)	22.9	21.5	0.127
Mean follow up, days (range)	523.7 (217-719)	606.9 (336-724)	0.071
Radiologic stage in JIC			
1	3 (16.7%)	0 (0%)	
2	8 (44.4%)	17 (77.3%)	
3A	7 (38.9%)	5 (22.7%)	
Locations of osteonecrosis in JIC			
Type C-1	8 (44.4%)	8 (36.4%)	
Type C-2	10 (55.6%)	14 (63.6%)	

*JIC* Japanese Investigation Committee

### Treatments

Teriparatide (20 μg) was subcutaneously administered once per day [23, 24] while alendronate (35 mg) was orally administered once per week [25]. In Japan, daily 20 μg teriparatide and weekly 35 mg alendronate administration is approved for the treatment of osteoporosis. None of the patients received vitamin D3, and non-steroid anti-inflammatory agents were given when needed to relief the pain.

### Assessment of collapse progression

**Fig. 1:**
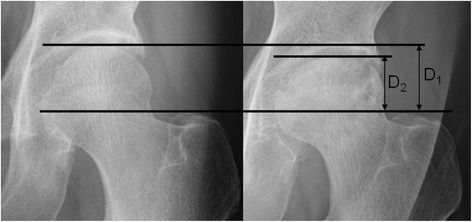
The progression of collapse of the femoral head of NOFH. The progression of collapse (D_1_-D_2_) before administration (*left*) and at every follow-up (*right*) using anteroposterior radiographs. The baseline is the top of the greater trochanter of the femur

Plain anteroposterior hip radiographs were taken prior to the treatment courses and at every interval of between three to six-month follow-up visit. The JIC staging and locations of osteonecrosis were estimated on X-ray examinations or MRI. Collapse progression of the femoral head was evaluated on anteroposterior hip radiographs in neutral rotation before administration and at every follow-up (Fig. 1) with the same imaging conditions, for avoidance of differences caused by magnification effects. Collapse progression with > 1 mm was defined as “advanced collapse”, and as “stable radiologic disease” when collapse progression was < 1 mm. The follow-up periods for the treatment courses were assigned for two years.

### Statistical analysis

Student’s *t*-test and the chi-square test was used to compare the pharmacological effects of the two groups. Normality of the samples were confirmed by Shapiro-Wilk test. Stable radiologic disease analysis was performed using Kaplan-Meier analysis. Inter-group comparison of Kaplan-Meier data was performed using the log-rank test. A *p*-value < 0.05 was deemed significant, as described in the figure legends. Analyses were performed using Ekuseru-Toukei 2010 (Social Survey Research Information Co., Ltd., Tokyo, Japan).

## Results

**Fig. 2:**
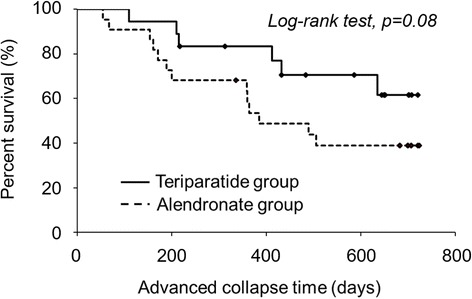
The Kaplan-Meier curves of the teriparatide group (*solid line*) and the alendronate group (*dotted line*) with advanced collapse as the end-point

**Fig. 3:**
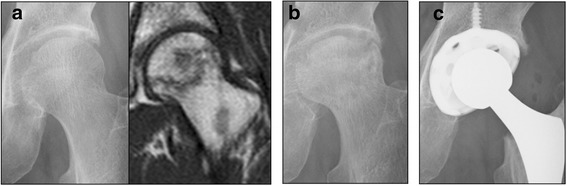
Case 1. Sixty-seven years old man with NOFH. **a** Anteroposterior radiograph and magnetic resonance imaging (T1WI) at the first examination showing NOFH. After diagnosis, alendronate was administered. **b** Collapse of the femoral head has progressed. **c** Eventually, THR was performed

**Fig. 4:**
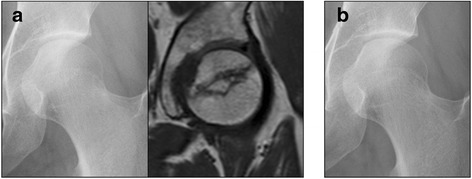
Case 2. Twenty-seven years old woman with NOFH. **a** Anteroposterior radiograph and magnetic resonance imaging (T1WI) at the first examination showing NOFH. After diagnosis, teriparatide was administered. **b** The femoral head showed no progression of collapse for one year and 8 months

To investigate the pharmacological usefulness of teriparatide for treatment of NOFH, a 6-months to 2-years follow-up study of 15 patients (18hips) who received teriparatide was performed. In parallel, same follow-up study of 17 patients (22 hips) who received alendronate was performed to compare the efficiencies of teriparatide. There were no complications observed on the patients during the period of study caused by the treatment course of teriparatide or alendronate. Notably, collapse progression was observed in 59.1% of patients received alendronate treatment, and in 33.3% of patients received teriparatide treatment, suggesting that teriparatide had a tendency to reduce collapse progression of femoral head (Table 2). Next, Kaplan-Meier curves were generated for patients with advanced collapse as the end-point (Fig. 2). Although there was no significant difference between the two treatments, patients treated by teriparatide exhibited a 6-month-prolonged radiologic disease (94.4% (90% CI 85.6 -100)) as compared to alendronate (77.3% (90% CI 62.6 – 92.0)). Moreover, a longer 1-year stability (83.3% (90% CI 68.9 – 97.8)) was noted in patients treated by teriparatide than that noted in patients treated by alendronate (53.6% (90% CI 35.8 – 71.3) (*p* = 0.08, log-rank test). Moreover, treatment with teriparatide resulted in a significant reduction in collapse progression (0.67 mm) as compared to that with alendronate (1.24 mm; *p* = 0.049) (Table 2). Representative case 1 is a 67-year-old man with NOFH, type C-2 in the JIC classification and JIC stage 2 that was treated by alendronate (Fig. 3). Collapse progression was 3.3 mm after 21 months of treatment course, and eventually THR was performed (Fig. 3). Representative case 2 is a 27-year-old woman with NOFH, type C-2 in the JIC classification and JIC stage 1 that was treated by teriparatide (Fig. 4). Collapse progression of the femoral head did not occur over a period of 20 months (Fig. 4). Taken together, our data suggest that teriparatide is promising therapeutic agent for NOFH.

**Table 2:** The occurrence rate of advanced collapse in the teriparatide and alendronate groups at the end of follow-up and the final collapse progression in the teriparatide and alendronate groups

	Teriparatide group	Alendronate group	*p* value
Advanced collapse	6/18 (33.3%)	13/22 (59.1%)	0.105
Final collapse progression	0.67 mm (0.00-3.61 mm)	1.24 mm (0.00-3.22 mm)	0.049^‡^

‡Significant

## Discussion

NOFH causes decreased vascular supply to the trabecular bone of the femoral head, resulting in collapse of articular surface of the femoral head and severe hip pain in young adults. The precise mechanism of collapse in NOFH has not been clarified; however, excessive bone resorption by osteoclast in necrotic regions is thought to induce collapse of the femoral head [7]. The disease is progressive and once collapse of the femoral head occurs, osteoarthritis of the hip joint is developed in few years. Most of cases need THR for relieving the severe hip pain caused by osteoarthritis; however, due to limited durability of THR, this procedure is not recommended for young adults. Therefore, conservative treatment that prevents collapse progression of the femoral head in early stage of NOFH is highly demanded.

Several pharmacological agents have been used for treatment of NOFH, including statins [26], anticoagulants [27, 28], prostacyclin [29, 30], and bisphosphonates. Bisphosphonates, namely alendronate, are the most common used [31]. Alendronate prevents early collapse of the femoral head in NOFH [32], and improves clinical function with better rate of collapse [11]. However, the frequently reported failures of alendronate in preventing collapse progression and its serious complications including osteonecrosis and atypical fractures [12, 33–38] highlight the need for more effective and safer therapeutic option for the treatment of NOFH.

We speculate that enhancing osteogenesis might contribute to the prevention of femoral head collapse progression. Teriparatide is known to increase the life-span of mature osteoblasts by preventing their apoptosis [39]. Teriparatide has been reported to increase cancellous bone volume and connectivity, and to improve trabecular morphology with a shift toward more plate-like structure [18]. Moreover, intermittent use of teriparatide exerts anabolic action on cortical bone with improved cancellous bone microarchitectures [40]. Recently, several case reports showed successful outcome of teriparatide for the treatment of osteonecrosis [18–20] and fracture-healing [41]. Consistently, our study showed that treatment with teriparatide resulted in lower rate of collapse progression than alendronate. Treatment with alendronate has been reported to be correlated with high incidence of collapse progression (65.6%) [12]. Likewise, in our study, the rate of head collapse in the alendronate-treated group fell within this range (59.1%), while lesser incidence was noted in teriparatide-treated patients (33.3%). This can be explained by the fact that teriparatide enhances osteoblast activity in the necrotic lesion, leading to an increase in cancellous bone volume and trabecular thickness of the femoral head.

This study has some limitations that must be pointed out. The mean follow-up period was longer in the alendronate group, and the patient’s average age of teriparatide group was 8 years younger than alendronate group. This may give an advantage of bone growth. The current study is retrospective study, covers small sample size (number of patients), doesn't include the clinical outcome or functional parameters post treatments, and doesn't consider the differences between radiological stage or location of osteonecrosis in the patients. Further study, including a larger number of samples with longer follow-up period is needed to conclusively demonstrate the therapeutic use of teriparatide for treatment of NOFH.

## Conclusion

This is the first study demonstrating that teriparatide is a potent conservative option for treatment of NOFH. The treatment course by teriparatide results in lesser collapse progression of the femoral head than that by alendronate, which is considered as the traditional pharmacological option for preventing collapse progression of the femoral head.

## Abbreviations

JIC: Japanese Investigation Committee
NOFH: Nontraumatic osteonecrosis of the femoral head
THR: Total hip replacement
